# 2185 The effects of autoimmune inflammation on proliferation, differentiation, and androgen receptor signaling in adult prostate stem cells

**DOI:** 10.1017/cts.2018.132

**Published:** 2018-11-21

**Authors:** Paula Cooper, Hsing-Hui Wang, Meaghan Broman, Emery Goossens, Hristos Kaimakliotis, Scott Crist, Nadia Atallah, Majid Kazemian, Bennett Elzey, Liang Cheng, Timothy L. Ratliff

**Affiliations:** 1 Indiana University School of Medicine; 2 University of North Carolina Chapel Hill; 3 Purdue University

## Abstract

OBJECTIVES/SPECIFIC AIMS: The primary goal of this project is to verify findings from a murine prostatitis model in the human setting. METHODS/STUDY POPULATION: Methods include primary cell isolation and culture, FACS, adoptive transfer, 3D cell culture, histology, immunofluorescence, xenograft, and tissue recombination. The study population includes patients undergoing HoLEP or radical prostatectomy due to hyperplasia or adjacent bladder or prostate cancer. RESULTS/ANTICIPATED RESULTS: Having verified similar sensitivities to androgen receptor (AR) inhibitors between naive murine and human basal prostate stem cells, we anticipate that autoimmune inflammation in humans affects the response of basal prostate stem cells in a manner similar to the murine setting as well. This includes increased proliferation, increased differentiation, and decreased response to AR inhibitors. DISCUSSION/SIGNIFICANCE OF IMPACT: The identification of survival mechanisms used by basal prostate stem cells in an androgen deprived environment may give insight to the process by which prostate cancer becomes androgen independent. The effect of inflammation on proliferation, survival, and AR signaling in these cells may also provide information relevant to cancer initiation and progression.

